# Finite-Time Thermodynamics of Battery Discharging: Power–Efficiency Trade-Off and Optimization

**DOI:** 10.3390/e28080852

**Published:** 2026-07-30

**Authors:** Rui-Han Liu, Yun-Qian Lin, Yu-Han Ma

**Affiliations:** 1 School of Physics and Astronomy, Beijing Normal University, Beijing 100875, China; 2 Key Laboratory of Multiscale Spin Physics (Ministry of Education), Beijing Normal University, Beijing 100875, China

**Keywords:** battery discharging, finite-time thermodynamics, power-efficiency trade-off, multistage constant-current discharging, battery management system, Pareto front

## Abstract

Battery discharging is governed by a fundamental trade-off between output power and energy conversion efficiency due to internal dissipation. In this paper, we demonstrate that such a trade-off universally yields a parabolic envelope P∝η(1−η). The efficiency at maximum power is exactly one half, mirroring the well-known half-Carnot limit in finite-time thermodynamics. To extend this bound into practical operational rules, we formulate a multistage constant-discharging (MSCD) schedule subject to simultaneous real-time load demands and a global discharging deadline. Analytical resolution via the Karush–Kuhn–Tucker conditions reveals a remarkably compact optimal policy: $I_{i}^{★}=\max\left(I_{i}^{\mathrm{req}},I_{0}\right)$. Under this rule, stages limited by external demand run exactly at their minimum required currents, while all remaining stages are elevated to a uniform baseline $I_{0}$ fixed by the deadline constraint. By tracing the dissipation–time Pareto front, we quantify how internal resistance shifts the operational boundaries and sharpens the trade-off corner. This analysis establishes a rigorous thermodynamic baseline for the scheduling layer of battery management systems, offering natural extensions to nonlinear models incorporating temperature and state-of-charge dependencies.

## 1. Introduction

To reduce dependence on fossil fuels and lower carbon emissions, clean energy has attracted increasing attention [1]. However, the intermittent nature of renewable energy sources poses a challenge for stable energy supply [2]. Batteries provide a practical solution to this challenge and have been widely used in electric vehicles, smartphones, and other electronic devices. Nevertheless, improper operation, such as over-charging [2], over-discharging [3], and high temperatures [4], can degrade battery performance and compromise safety. Therefore, effective battery management is crucial for extending battery lifetime and improving operational safety and efficiency. Massive efforts have been dedicated to enhancing battery performance, encompassing novel battery designs [5,6,7], advanced battery materials [8,9,10,11], and optimized charging/discharging strategies [12,13,14].

The core of battery operation lies in these charging and discharging cycles. In practice, Battery Management Systems (BMSs) [15] schedule these cycles by tracking observable properties such as cell voltage, current, and temperature. However, existing BMS models inevitably face a practical compromise between physical precision and computational feasibility [1,2]: detailed electrochemical models are computationally prohibitive, equivalent-circuit models require constant parameter recalibration against shifting dynamics [16], and data-driven approaches risk out-of-distribution failure. To overcome these model-dependent compromises and uncover universal performance boundaries, finite-time thermodynamics (FTT) provides a physical, tractable framework [17,18,19,20,21,22,23,24]. At its core, FTT establishes that driving energy transfer at a finite rate invariably incurs irreversible dissipation. In a battery, this manifests as a direct competition: extracting stored electrochemical energy at a larger current accelerates power delivery, but induces quadratic Joule heat across the internal resistance, thereby suppressing the discharge efficiency η. This trade-off serves as the exact electrochemical analogue of the typical constraint relations analyzed in finite-time thermodynamic cycles [25,26,27,28,29,30,31,32,33,34,35,36,37,38,39,40,41].

Recently, two of the present authors and their collaborators successfully extended the FFT perspective to battery charging [42]. By deriving the exact thermodynamic power–efficiency trade-off for RC circuits, they provided a theoretical foundation for empirical multistage constant-current (MSCC) protocols [43,44,45] and determined optimal schedules through time minimization. A corresponding FFT analysis for the discharging process, however, remains absent. Unlike charging, discharging cannot be treated as a simple time-reversed step. While a charger actively tunes the input current to suppress heat, discharging is strictly load-driven [46] and tightly constrained by external power demands alongside instantaneous voltage, current, and state-of-charge (SOC) limits [47]. Delivering high power requires large currents, which inevitably exacerbate internal dissipation and spatial nonuniformity, driving the cell prematurely to its cutoff potential and shrinking its usable capacity [48]. Furthermore, repeated high-current pulses accelerate internal resistance growth and battery degradation [49,50]. Since practical BMSs track these degradation states through the aforementioned model approximations, the fundamental thermodynamic boundary governing the discharging process itself remains undefined.

In this paper, we bridge this gap by applying the FTT approach to battery discharging. To unveil the underlying physical constraints, we address two core questions: (i) does a battery discharging under a time-varying load obey a universal thermodynamic bound? (ii) can this bound yield a closed-form optimal scheduling rule? This paper is organized as follows. In Section 2, we examine three discharging models of increasing realism, encompassing constant electromotive force, finite-capacitance RC dynamics, and active constant-current control. Across all three models, we show that the battery discharging obeys the same parabolic envelope P∝η(1−η), with the efficiency at maximum power equal to exactly one half. We then compare active constant-current (CC) discharge with passive resistive discharge at the same voltage window and total discharge time, showing that active control gives a lower internal heat loss. Next, Section 3 introduces a multistage constant-current discharging (MSCD) schedule and solves the optimization analytically using the Karush–Kuhn–Tucker (KKT) conditions. The optimum has a simple structure: demand-limited stages operate at their minimum required currents, while the remaining stages share a uniform baseline current set by the global deadline. We then characterize the feasible operating regimes imposed by efficiency and load-power bounds, construct the dissipation–time (*Q*–τ) Pareto front, and extend the analysis to the internal-resistance parameter space. In addition, to test the robustness of results obtained in Section 2 and Section 3 beyond the RC circuit, we further include a series inductance in the equivalent circuit. It is shown that inductance mainly affects transient dynamics, switching voltage constraints, and local branch stability, while the steady-state power–efficiency parabola and the MSCD optimization structure remain unchanged. Finally, Section 5 concludes the results and discusses possible extensions.

## 2. Power–Efficiency Parabola of Battery Discharging

### 2.1. Infinite-Capacitance Regime: Constant Electromotive Force and Matched-Load Limit

**Figure 1:**
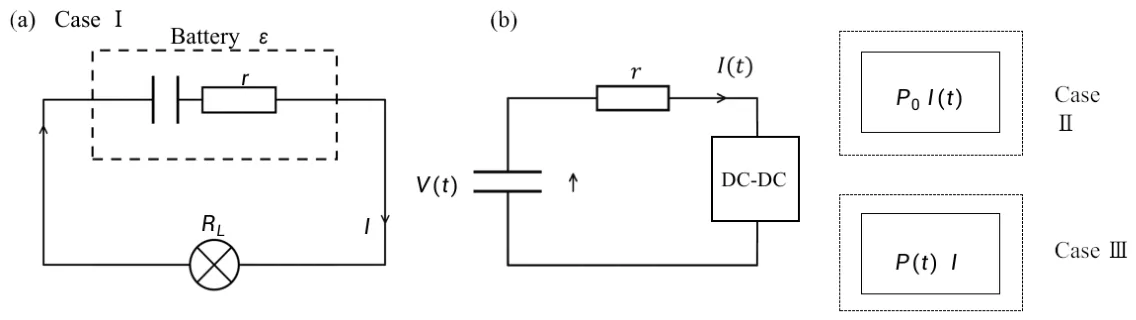
Equivalent-circuit schematics for the discharge models. (**a**) The constant electromotive force model, with an ideal source in series with internal resistance *r* driving a resistive load $R_{L}$. Middle: The finite-capacitance model, where a capacitor with internal resistance *r* is coupled to a DC-DC converter that regulates the cell current I(t). (**b**) The prescribed external conditions used in the analysis, namely fixed output power $P_{0}$ or fixed current *I*.

We begin with the simplest discharging model, as illustrated in Figure 1a. The battery is modeled as an effectively infinite capacitance in series with an internal resistance *r*, ensuring that its internal electromotive force ε remains strictly constant throughout the entire discharging process. When supplying an external resistive load $R_{L}$, Kirchhoff’s voltage law and energy conservation dictate that the load power is given by

(1) $$P\left(I\right)=\varepsilon I-I^{2}r,$$

where εI is the total chemical power of the battery and $I^{2}r$ is the dissipative power due to circuit heating. Thus, the discharging efficiency is the ratio of load power to the total chemical power supplied by the battery, namely

(2) η=P/(εI)=1−Ir/ε.

Combining Equations (1) and (2) and eliminating *I* yields

(3) $$P\left(\eta \right)=\frac{\varepsilon^{2}}{r}\;\eta \left(1-\eta \right).$$

Equation (3) shows that the output power is a concave parabola as a function of efficiency η, vanishing at the no-load point (η=1,I=0) and the short-circuit point (η=0,I=ε/r). The efficiency at maximum power and the corresponding peak power $P_{\max}$ are achieved when ∂P/∂η=0, yielding

(4) $$\eta_{\mathrm{EMP}}=\frac{1}{2},\;P_{\max}=\frac{\varepsilon^{2}}{4r}.$$

$P_{\max}$ is the largest instantaneous power that the battery can supply to any external load. It is attained when the load resistance is exactly the internal resistance *r*, with the corresponding optimal current being $I^{★}=\varepsilon /\left(2r\right)$. At the EMP point, exactly one half of the chemical power flux is dissipated as Joule heat; this fraction is independent of the electrochemistry and is the minimum dissipation the battery incurs. The battery result $\eta_{\mathrm{EMP}}=1/2$ has the same structure as the linear-response result for heat engines, where the efficiency at maximum power is one half of the Carnot bound, $\eta_{\mathrm{EMP}}=\eta_{C}/2$ [26,29]. When operating at maximum power, half of the energy flux is inevitably dissipated as Joule heat, which can strongly affect battery performance by promoting thermal runaway and accelerating aging under high discharge currents [51,52].

### 2.2. Finite-Capacitance Regime: Two-Branch Structure and Instantaneous Power Limit

In Section 2.1, the current is strictly constrained by ε and the load resistance $R_{L}$, namely $I=\varepsilon /(r+R_{L})$. In practice, the external device usually requires a prescribed power $P_{0}$. To meet this demand, the battery is typically interfaced with the device through a power-electronic converter, such as a DC–DC converter, inverter, or motor controller, which actively regulates the current I(t) drawn from the battery.

Therefore, we replace the constant source ε by a finite capacitor *C* with voltage V(t) decreasing from $V\left(0\right)=V_{0}$ (Figure 1b). For a fixed output power $P_{0}$, the instantaneous power balance and charge conservation are

(5) $$V\left(t\right)I\left(t\right)=P\left(t\right)+rI^{2}\left(t\right),\;I\left(t\right)=-C\frac{dV(t)}{dt},$$

where *r* is the internal resistance of the battery. With $P\left(t\right)=P_{0}$, a real solution of the power balance in Equation (5) exists only when $V^{2}-4rP_{0}\geq 0$, setting the instantaneous power limit

(6) $$P_{0}\leq P_{\max}\left(t\right)=\frac{V^{2}\left(t\right)}{4r},$$

taking the same form as $P_{\max}$ in Equation (4). For $0<P_{0}<P_{\max}\left(t\right)$, the solution for the current is given by

(7) $$I^{\pm }\left(t\right)=\frac{V\left(t\right)\pm \sqrt{V^{2}\left(t\right)-4rP_{0}}}{2r}.$$

The instantaneous discharging efficiency η=P/(VI)=1−rI/V follows directly from Equation (5). Substituting $I_{\pm }$ into this relation yields

(8) $$\eta^{\mp }\left(t\right)=\frac{1\pm \sqrt{1-p}}{2},\;p\equiv \frac{P_{0}}{P_{\max}\left(t\right)}=\frac{4rP_{0}}{V^{2}\left(t\right)},$$

where p∈[0, 1] is the dimensionless instantaneous power. Inverting the efficiency reveals that

(9) p=4η(1−η),

which is identical in form to Equation (3). Furthermore, the low-current branch $I^{-}$ has $\eta^{+}>1/2$, while the high-current branch $I^{+}$ has $\eta^{-}<1/2$. Although the two current branches can supply the load power, the low-current branch is preferred because it dissipates less and operates at a lower C-rate, both favorable for cycle life [52,53]. At $P_{0}=P_{\max}\left(t\right)$ the branches merge at I=V/(2r). Since $P_{\max}\left(t\right)\propto V^{2}\left(t\right)$, the deliverable peak power decreases with SOC. It is worth emphasizing that $\eta_{\mathrm{EMP}}=1/2$ refers to the maximum-power efficiency of the idealized uncontrolled circuit shown in Figure 1. In practical discharge, battery management strategies usually impose additional constraints, such as limits on the current [54] and voltage drop [55]. These constraints typically keep the battery on the high-efficiency, low-current branch, rather than operating it exactly at the maximum-power point.

### 2.3. Constant-Current Discharging and Universality of the Parabola

Constant-current (CC) discharging is a basic strategy in battery use and BMS capacity testing [2,55], which can be implemented by converter control. Here, we consider a finite battery with a constant-current demand undergoing a full discharging process from $V_{0}$ to the cut potential $V_{f}$ [56]. With charge conservation, the total discharge time $t_{f}=C\left(V_{0}-V_{f}\right)/I$ and the instantaneous potential $V\left(t\right)=V_{0}-\left(I/C\right)t$ are maintained.

Moreover, the released free energy during discharging follows $\Delta E=C(V_{0}^{2}-V_{f}^{2})/2$, while the cumulative Joule heat is $Q=rIC(V_{0}-V_{f})$. According to the first law of thermodynamics, $W_{\mathrm{out}}=\Delta E-Q$ is obtained as

(10) $$W_{\mathrm{out}}=C\left(V_{0}-V_{f}\right)\left(\frac{V_{0}+V_{f}}{2}-rI\right).$$

To quantify the thermodynamic performance of the discharging process, we introduce the discharging efficiency $\bar{\eta }\equiv W_{\mathrm{out}}/\Delta E$ and discharging power $\bar{P}=W_{\mathrm{out}}/t_{f}$, which, respectively, read

(11) $$\bar{\eta }\equiv 1-\frac{rI}{\bar{V}},\;\bar{P}\left(I\right)=C\frac{V_{0}-V_{f}}{t_{f}}\left(\frac{V_{0}+V_{f}}{2}-rI\right),$$

where $V\equiv \frac{V_{0}+V_{f}}{2}$. Substituting $I=\left(\bar{V}/r\right)\left(1-\bar{\eta }\right)$ into $\bar{P}\left(I\right)$ gives

(12) $$\bar{P}\left(\bar{\eta }\right)=\frac{{\bar{V}}^{2}}{r}\;\bar{\eta }\left(1-\bar{\eta }\right).$$

Thus, the CC process has the same parabola as Equation (3), with ε replaced by the mean open-circuit voltage $\bar{V}=\left(V_{0}+V_{f}\right)/2$.

**Figure 2:**
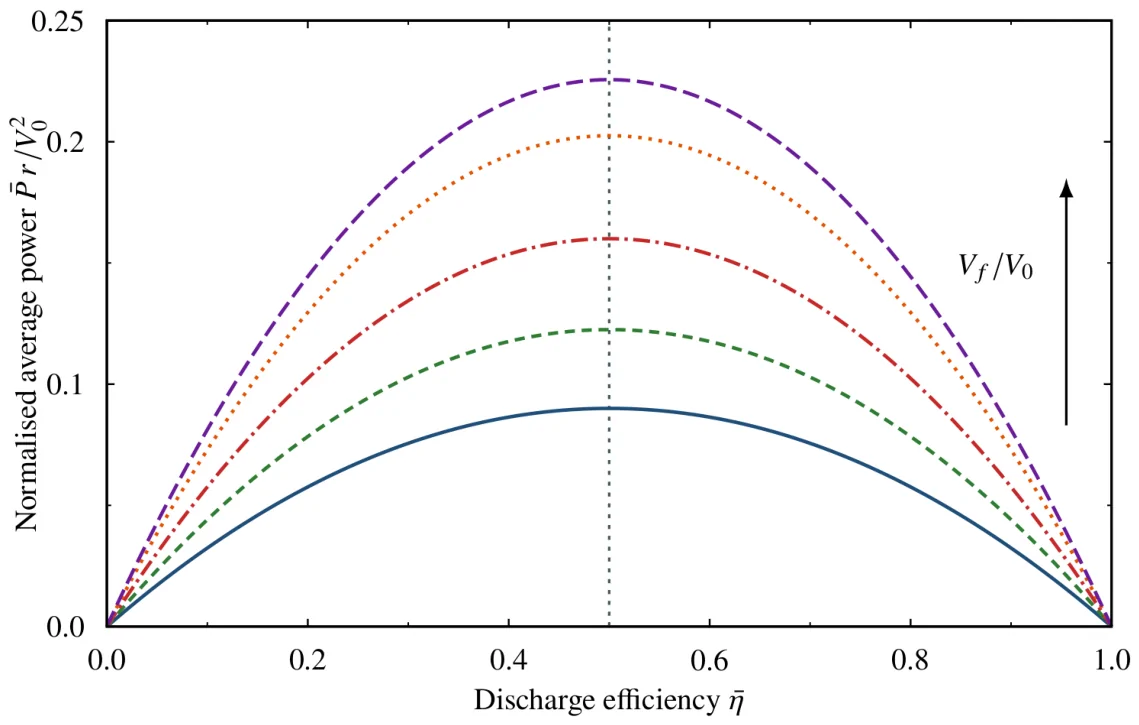
Normalized average power $\bar{P}\;r/V_{0}^{2}$ versus process-averaged efficiency $\bar{\eta }$ for constant-current discharging at five cut-off ratios from deep to shallow discharge. From bottom to top, the curves correspond to $V_{f}/V_{0}=0.20,\;0.40,\;0.60,\;0.80,$ and 0.90. The dotted vertical line marks the maximum-power efficiency $\bar{\eta }=1/2$.

Figure 2 plots Equation (12) with a series of cut-off ratios $V_{f}/V_{0}$. It is obvious that the curves exhibit the same parabolic dependence on $\bar{\eta }$: the curves vanish at $\bar{\eta }=0$ and 1, and peak at $\bar{\eta }=1/2$. As $V_{f}/V_{0}$ increases, the curve is lifted due to the larger vertical scale ${\bar{V}}^{2}/r$. Therefore, a shallower discharge permits a higher average power at a fixed efficiency, which is consistent with the performance improvement observed in pulsed-discharge protocols [57]. In the limit $V_{f}/V_{0}\to 1$, the peak approaches the constant-EMF value with $\varepsilon =V_{0}$.

The emergence of the identical parabolic envelope P∝η(1−η) across all three models discussed in the current section reveals a robust thermodynamic universality. It establishes that the efficiency at maximum power is exactly η=1/2. Because the theoretical reversible limit for battery discharging is unity ($\eta_{\mathrm{rev}}=1$), this optimum represents exactly half of the reversible bound, mapping rigorously to the well-known $\eta_{C}/2$ efficiency at maximum power (the half-Carnot limit) in finite-time heat engines [23,24,26,27,29,30]. This yields a fundamental takeaway: while internal chemistry and external control of the battery dictate the absolute power scale, the normalized parabolic envelope remains an invariant signature of Ohmic dissipation.

We close this section with a remark on the scope and limitations of the foundational model. The equivalent-circuit model adopted here serves as a minimal analytical baseline to derive the thermodynamic bounds and formulate the MSCD optimization. By using a finite capacitance to represent the SOC-dependent open-circuit voltage, the model captures the inherent trade-off between average power and extractable energy [58]. While real batteries involve complex polarization, diffusion, and entropy–heat dynamics that necessitate higher-order or electrochemical models [54,59,60,61,62,63], we deliberately isolate the primary Ohmic-loss mechanism responsible for the parabolic envelope, leaving these advanced integrations for future work. Furthermore, this baseline temporarily omits series inductance. Because the MSCD protocol operates via piecewise constant-current stages (dI/dt≈0), inductive effects strictly vanish during bulk discharging and only perturb the brief switching transients without altering the stage-wise Joule dissipation. A rigorous discussion of these inductive effects is deferred to Section 4.

### 2.4. Performance of CC Discharging

In the passive circuit, no converter is present to regulate the current or output power. The battery simply discharges through a fixed external load $R_{L}$, leading to the uncontrolled RC relaxation

(13) $$V\left(t\right)=V_{0}e^{-t/[\left(r+R_{L}\right)C]},$$

where the capacitor is finite. By contrast, in CC discharge, a power-electronic controller is introduced to enforce a prescribed current profile. Here, we compare the performance of CC discharging with passive discharging under a fixed total discharge time $t_{f}$ over the same voltage window $\left[V_{f},\;V_{0}\right]$.

Fixing $V_{f}$ yields the total resistance in the circuit $r+R_{L}=t_{f}/\left[C\ln\left(V_{0}/V_{f}\right)\right]$, with which the dissipation induced by the internal resistance *r* is given by

(14) $$Q_{\mathrm{diss},\mathrm{passive}}=\frac{1}{2}C\left(V_{0}^{2}-V_{f}^{2}\right)\;\frac{rC\ln(V_{0}/V_{f})}{t_{f}}.$$

Under the constraint of charge $C(V_{0}-V_{f})$, the current in CC discharge is, thus, $I=C\left(V_{0}-V_{f}\right)/t_{f}$, revealing that the internal dissipation

(15) $$Q_{\mathrm{diss},\mathrm{active}}=\frac{C^{2}r{\left(V_{0}-V_{f}\right)}^{2}}{t_{f}}.$$

**Figure 3:**
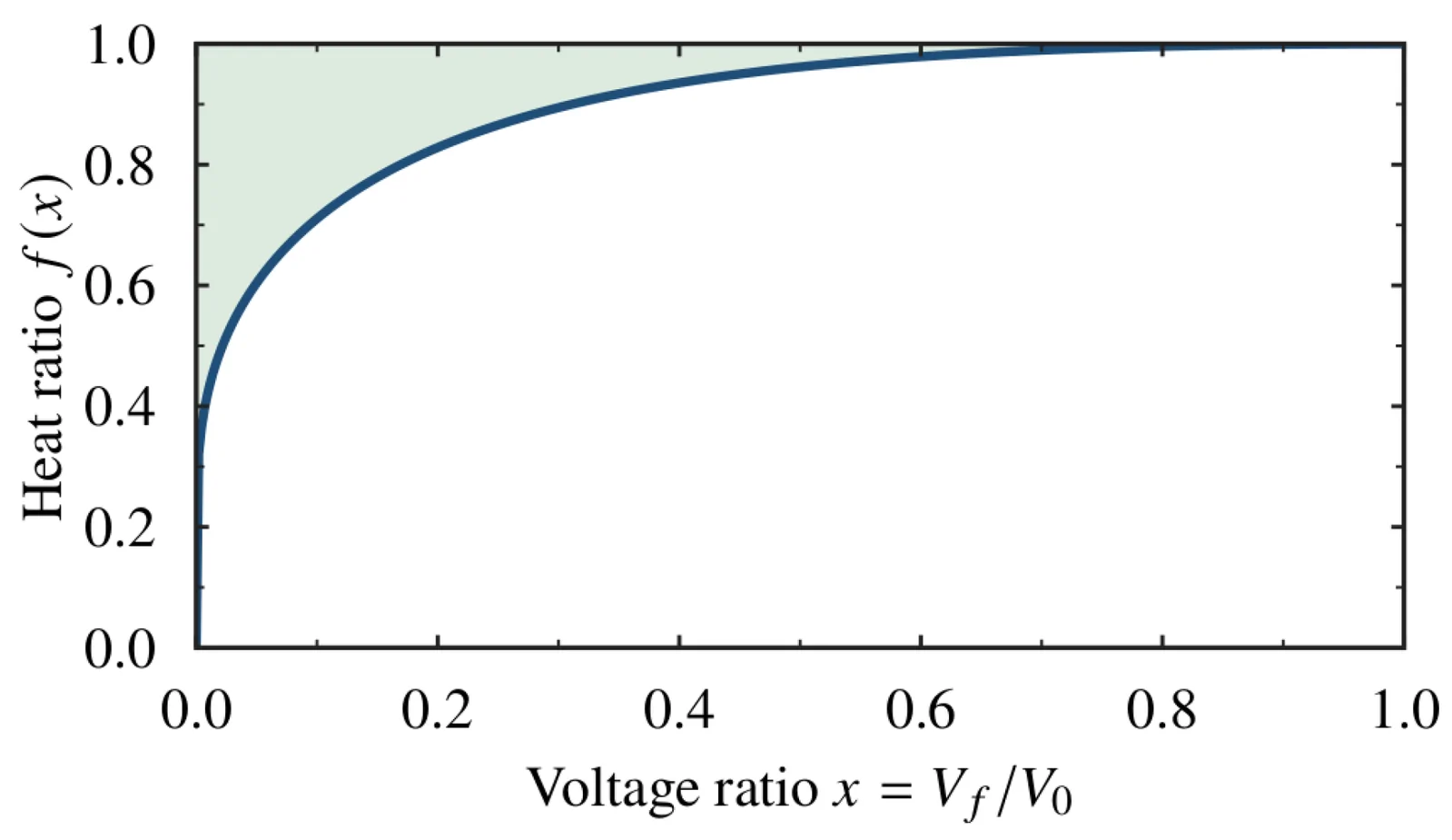
Heat ratio $f\left(x\right)=Q_{\mathrm{diss},\mathrm{active}}/Q_{\mathrm{diss},\mathrm{passive}}$ is the function of the voltage ratio $x=V_{f}/V_{0}$. The green region is the saving fraction of dissipation 1−f.

We quantify the performance of the active control by

(16) $$f\left(x\right)\equiv \frac{Q_{\mathrm{diss},\mathrm{active}}}{Q_{\mathrm{diss},\mathrm{passive}}}=\frac{2(1-x)}{\left(x+1)\ln(1/x\right)},$$

where $x=V_{f}/V_{0}$ is the voltage ratio and (1−x) means the depth of discharge in the battery. Figure 3 plots f(x), which remains below unity over the whole range of *x*. In other words, active CC control always dissipates less heat than passive discharge under the same voltage window and discharge time.

## 3. Multistage Constant-Current Discharging (MSCD)

Section 2.2 shows that, for a prescribed power demand *P*, operation on the high-efficiency branch imposes a minimum admissible current $I^{-}$. Increasing the current can accelerate the discharging, but only at the expense of enhanced Ohmic dissipation and reduced efficiency. Therefore, fast discharge naturally becomes a constrained optimization problem: the current should be increased to shorten the discharge time, while remaining within the admissible range set by the power demand and efficiency constraint.

Motivated by the multistage constant-current (MSCC) protocol widely used in charging [43,44,45], we introduce a multistage constant-current discharging (MSCD) protocol. In this protocol, the discharge process is divided into stages, and a constant current $I_{i}$ is assigned to each stage while satisfying the stage-wise requirement $I_{i}\geq I_{i}^{-}$. The resulting problem is not a simple time reversal of charging: the load-power demand imposes lower bounds on the stage currents, while all stages are coupled through the global discharge-time constraint. The optimal MSCD schedule is, therefore, obtained by solving the Karush–Kuhn–Tucker (KKT) conditions [64,65], as detailed in Section 3.2.

### 3.1. Stage-Wise Description and the Unconstrained Minimum-Dissipation Bound

We partition the voltage window $\left[V_{f},\;V_{0}\right]$ into *N* stages by the levels $V_{0}>V_{1}>\cdots >V_{N}=V_{f}$. The initial voltage and the discharging current in the *i*th stage are $V_{i-1}$ and $I_{i}$. The discharging time $\Delta t_{i}$, the released charge $\Delta q_{i}$, and the stage Joule heat $Q_{i}$ are, respectively, given by:

(17) $$\begin{array}{cc} \Delta t_{i} & =\frac{C(V_{i-1}-V_{i})}{I_{i}}, \end{array}$$

(18) $$\begin{array}{cc} \Delta q_{i} & =C(V_{i-1}-V_{i}), \end{array}$$

(19) $$\begin{array}{cc} Q_{i} & =I_{i}^{2}\;r\;\Delta t_{i}=rC\left(V_{i-1}-V_{i}\right)I_{i}. \end{array}$$

Therefore, the total discharge time τ and the total dissipated Joule heat *Q* are

(20) $$\begin{array}{cc} \tau & =\sum_{i=1}^{N}\frac{C(V_{i-1}-V_{i})}{I_{i}}, \end{array}$$

(21) $$\begin{array}{cc} Q & =\sum_{i=1}^{N}rC\left(V_{i-1}-V_{i}\right)I_{i}. \end{array}$$

Supposing only $V_{0}$, $V_{f}$ and the total time τ are prescribed and *r*, *C* are constants, the Cauchy–Schwarz inequality gives $Q\tau \geq {\left[rC^{2}\sum_{i}\left(V_{i-1}-V_{i}\right)\right]}^{2}=rC^{2}{\left(V_{0}-V_{f}\right)}^{2}$. Equality holds if all $I_{i}$ are equal, namely a single-current discharge. This is the discrete-stage counterpart of the Salamon–Berry thermodynamic-length result [21,66] and sets the baseline against which the constrained optimum of Section 3.2 is measured.

### 3.2. Optimal Protocol Under Power and Deadline Constraints

Although the protocol is now multistage, each stage individually is still governed by the discussion in Section 2.2. Evaluating the high-efficiency branch of Equation (7) at $V=V_{i}$ and $P_{0}=P_{i}^{\mathrm{req}}$ gives

(22) $$I_{i}^{-}=\frac{V_{i}-\sqrt{V_{i}^{2}-4rP_{i}^{\mathrm{req}}}}{2r},\;P_{i}^{\mathrm{req}}\leq \frac{V_{i}^{2}}{4r}.$$

The MSCD optimization problem is then

(23) $$\begin{array}{cc} \min_{\left\{I_{i}\right\}}\; & \sum_{i=1}^{N}rC\left(V_{i-1}-V_{i}\right)I_{i},\\ s.t.\; & \sum_{i=1}^{N}\frac{C(V_{i-1}-V_{i})}{I_{i}}\leq \tau_{\max},\;I_{i}\geq I_{i}^{-}. \end{array}$$

MSCD differs from MSCC through its stage-wise lower bound. In the MSCC treatment of Lei et al. [42], the free stage currents are obtained by minimizing the total charging time, yielding $I_{i}^{2}=I_{i-1}I_{i+1}$ and, hence, a decreasing current sequence with equal stage time. However, in MSCD, each stage must satisfy the lower bound $I_{i}\geq I_{i}^{-}$. Because it is not known in advance which lower bounds will bind, the optimum must be determined from the KKT complementary-slackness conditions, leading to the two groups in Equation (26).

To incorporate the two constraints, we introduce a Lagrange multiplier λ≥0 for the global discharge deadline and multipliers $\mu_{i}\geq 0$ for each lower bound $I_{i}\geq I_{i}^{-}$. The Lagrangian is

(24) $$\begin{array}{cc} L=\; & \sum_{i}r\Delta q_{i}I_{i}+\lambda \;\left(\sum_{i}\frac{\Delta q_{i}}{I_{i}}-\tau_{\max}\right)-\sum_{i}\mu_{i}\left(I_{i}-I_{i}^{-}\right), \end{array}$$

where the stage charge $\Delta q_{i}=C\left(V_{i-1}-V_{i}\right)$ from Equation (18), and the total heat and time simplify to $Q=\sum_{i}r\Delta q_{i}I_{i}$ and $\tau =\sum_{i}\Delta q_{i}/I_{i}$.

The KKT stationarity condition applies to each stage current: $\partial L/\partial I_{i}=0$, revealing

(25) $$I_{i}^{2}=\frac{\lambda /r}{1-\mu_{i}/\left(r\Delta q_{i}\right)}.$$

The complementary-slackness condition, $\mu_{i}\left(I_{i}-I_{i}^{-}\right)=0$, encodes the active-bound structure of the lower-current constraint. For $\mu_{i}=0$, the stage shares the common current $I_{0}\equiv \sqrt{\lambda /r}$. When $\mu_{i}>0$, the lower bound is active, enforcing $I_{i}=I_{i}^{-}$, namely, the stage current is fixed at its minimum admissible value. Combining the two cases gives

(26) $$I_{i}^{★}=\max\;\left(I_{i}^{-},\;I_{0}\right),$$

where $I_{0}\equiv \sqrt{\lambda /r}$ is shared by all unpinned stages, while pinned stages remain at $I_{i}^{-}$. With the global discharge deadline, $I_{0}$ is governed by

(27) $$\sum_{i=1}^{N}\frac{C(V_{i-1}-V_{i})}{\max(I_{i}^{-},\;I_{0})}=\tau_{\max}.$$

Since the left-hand side decreases monotonically with $I_{0}$, $I_{0}$ can be efficiently obtained by bisection. Substituting $I_{i}^{★}$ into the heat loss, we obtain

(28) $$Q_{\mathrm{MSCD}}\left(\tau_{\max}\right)=\sum_{i=1}^{N}rC\left(V_{i-1}-V_{i}\right)\;I_{i}^{★}\left(\tau_{\max}\right).$$

**Figure 4:**
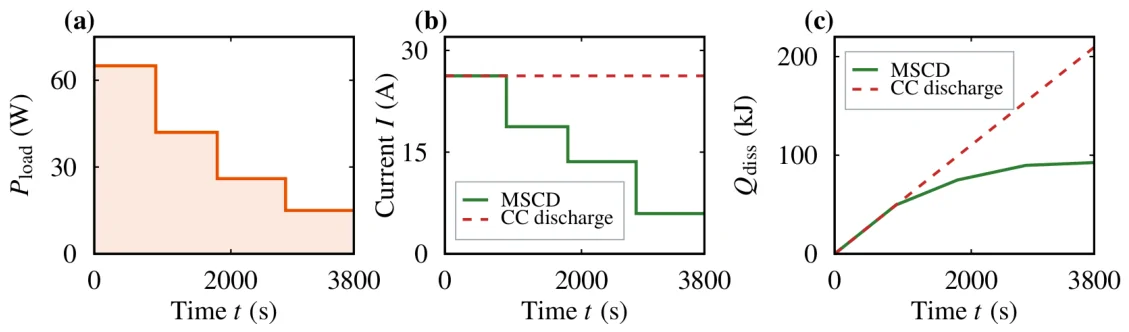
Comparison of two discharging protocols under a stepwise dynamic load. (**a**) The prescribed stepwise load-power demand $P_{\mathrm{load}}\left(t\right)$ (orange shaded region). (**b**) The discharging current applied in MSCD (green solid line) and CC discharge (red dashed line). (**c**) Accumulated Joule heat $Q_{\mathrm{diss}}\left(t\right)$ during discharging.

Figure 4 compares the optimized MSCD protocol with conventional CC discharge. The load demand is prescribed in a stepwise manner and remains constant within each stage, as shown in Figure 4a. To meet this demand, the MSCD protocol assigns a stage-dependent constant current, whereas the CC protocol applies the peak required current throughout the entire discharge process, as shown in Figure 4b. The corresponding accumulated heat losses are plotted in Figure 4c. Compared with CC discharge, for which $Q=rIC(V_{0}-V_{f})$, the MSCD protocol gives the stage-wise result in Equation (28) and reduces the total dissipation by lowering the current with lower load demands. This result shows that adapting the current to the stage-wise load demand suppresses unnecessary Joule heating while still satisfying the required power output.

As a practical five-stage example, we use the positive discharge-power steps of the DOE/INL PHEV charge-depleting cycle-life test profile [67]. The system-level powers are $P_{\mathrm{sys},i}=\left\{50,\;45,\;28.125,\;22.5,\;11.25\right\}\;\mathrm{kW}$. Following the Battery Size Factor (BSF) scaling rule in the same manual, we take BSF=1400 and obtain the stage load on a single battery demanding

(29) $$P_{i}^{\mathrm{req}}=\frac{P_{\mathrm{sys},i}}{\mathrm{BSF}}=\left\{35.71,\;32.14,\;20.09,\;16.07,\;8.04\right\}\;W.$$

We use the representative voltage grid $(V_{0},\;V_{1},\;\ldots ,\;V_{5})=4.2,\;4.0,\;3.75,\;3.5,\;3.25,\;3.0\;V$, together with an internal resistance r=0.08Ω [54,59]. The effective capacitance is set to $C=1.103\times 10^{4}\;F$, corresponding to an effective capacity of 3.68Ah over the voltage window 4.2−3.0V. For these parameters, Equation (22) gives the minimum admissible currents $I_{i}^{-}=11.64,\;11.29,\;6.80,\;5.76,\;2.90\;A$. If each stage operates at its lower bound, the resulting load-following schedule takes $\tau_{\mathrm{req}}≃37.8\;\min$. To illustrate the deadline-constrained optimization, we impose a tighter total discharge time, $\tau_{\max}=21.94\;\min$, corresponding to the Pareto-knee point determined later in Section 3.4. Solving Equation (27) yields $I_{0}≃9.37\;A$. Therefore, the first two stages remain pinned to $I_{i}^{-}$, while the last three are raised to $I_{0}$, giving $I_{i}^{★}=\left\{11.64,\;11.29,\;9.37,\;9.37,\;9.37\right\}\;A$. Substituting these currents into Equation (28) gives $Q_{\mathrm{MSCD}}≃10.75\;\mathrm{kJ}$ and $\eta_{\mathrm{MSCD}}≃77.4\%$. Compared with the single-peak-current benchmark, which holds $\max_{i}I_{i}^{-}$ throughout all stages, MSCD reduces Joule heat from 12.32kJ to 10.75kJ, corresponding to a 12.8% reduction, and raises the efficiency from 74.1% to 77.4%.

### 3.3. Feasibility Under Efficiency and Load Bounds

The same KKT analysis also applies when the constraint is placed on instantaneous efficiency. For a finite-capacitance battery, when no external power demand is imposed, a natural alternative optimization problem is to minimize dissipation over a prescribed discharge time while enforcing a lower bound $\eta_{0}$ on the instantaneous efficiency. In an MSCD stage, the instantaneous efficiency is given by

(30) $$\eta_{i}\left(V\right)=1-\frac{rI_{i}}{V}.$$

Since *V* decreases within the stage, the minimum efficiency occurs at the stage end $V_{i}$. The constraint $\eta_{i}\left(V_{i}\right)\geq \eta_{0}$, therefore, yields the stage-wise current upper bound

(31) $$I_{i}\leq I_{i}^{\eta }=\frac{\left(1-\eta_{0}\right)V_{i}}{r}.$$

Repeating the KKT analysis gives the analogous form

(32) $$I_{i}^{★}=\min\left(I_{c},\;I_{i}^{\eta }\right),$$

where the common current $I_{c}$ is governed by

(33) $$\sum_{i=1}^{N}\frac{C(V_{i-1}-V_{i})}{\min(I_{c},\;I_{i}^{\eta })}=\tau_{\max}.$$

Thus, the efficiency constraint sets the upper bound on the stage-wise current, whereas the load-power constraint in Equation (26) sets the lower bound.

For a fixed efficiency lower limit $\eta_{0}$, the current upper bounds $I_{i}^{\eta }$ are fixed, and changing the deadline only changes the common current $I_{c}$ in Equation (33). The shortest feasible discharge occurs when all stages are driven at their upper bounds,

(34) $$\tau_{\min}^{\eta }=\sum_{i=1}^{N}\frac{C(V_{i-1}-V_{i})}{I_{i}^{\eta }}.$$

If $\tau_{\max}<\tau_{\min}^{\eta }$, the discharge is infeasible. As $\tau_{\max}$ increases, $I_{c}$ decreases: just above $\tau_{\min}^{\eta }$, low-voltage stages are held at their upper bounds, while for a loose deadline, all upper bounds become inactive and the solution reduces to ordinary constant-current discharge.

**Figure 5:**
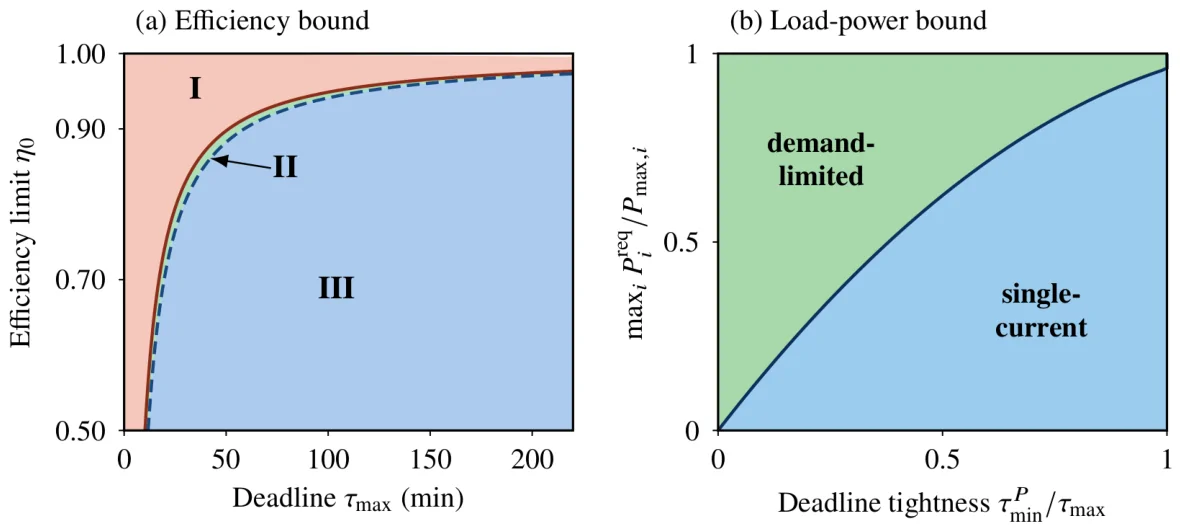
Feasibility maps for the two stage-wise bounds in MSCD. (**a**) A minimum instantaneous efficiency $\eta_{0}$ sets current upper bounds $I_{i}^{\eta }$. The solid and dashed curves denote $\tau_{\max}=\tau_{\min}^{\eta }$ and $I_{c}=\min_{i}I_{i}^{\eta }$, respectively. They divide the plane into the infeasible region (I), bounded-MSCD region (II), and CC region (III). (**b**) In the load-power-constrained case, prescribed stage powers set current lower bounds $I_{i}^{-}$. The axes are $\max_{i}P_{i}^{\mathrm{req}}/P_{\max,i}$ and $\tau_{\min}^{P}/\tau_{\max}$, and the curve $I_{0}=\max_{i}I_{i}^{-}$ separates the demand-limited MSCD region from the single-current region.

This behavior is summarized in Figure 5a, where the efficiency lower limit $\eta_{0}\in \left[0.5,\;1\right)$ is plotted against the deadline $\tau_{\max}$ for the high-efficiency range considered here. The solid curve gives the minimum feasible time, $\tau_{\max}=\tau_{\min}^{\eta }$, for a given $\eta_{0}$ so that region I is infeasible. The dashed curve is the upper-bound inactivity bound, $I_{c}=\min_{i}I_{i}^{\eta }$, at which the constrained MSCD solution reduces to CC discharge. Region II is the efficiency-bounded MSCD region between the two curves, where at least one low-voltage stage is held at its current upper bound. Region III is the CC region, where the common current is below all stage current upper bounds.

The load-constrained case has the opposite structure, because the prescribed stage powers set current lower bounds rather than upper bounds. For fixed load demands $P_{i}^{\mathrm{req}}$, the lower bounds $I_{i}^{-}$ are fixed. A shorter deadline requires a larger common current $I_{0}$, whereas a longer deadline allows a smaller $I_{0}$. The shortest feasible time is obtained when every stage is driven at the maximum-power current $V_{i}/\left(2r\right)$,

(35) $$\tau_{\min}^{P}=\sum_{i=1}^{N}\frac{C(V_{i-1}-V_{i})}{V_{i}/\left(2r\right)}.$$

If $\tau_{\max}<\tau_{\min}^{P}$, the discharge is infeasible. As $\tau_{\max}$ increases, $I_{0}$ decreases, so the solution moves from the single-current region, where all stages share the same current, to the demand-limited region, where stages with $I_{i}^{-}>I_{0}$ remain at their lower bounds and the remaining stages share $I_{0}$.

This behavior is summarized in Figure 5b. The map uses the five-stage load profile introduced in Section 3.2, with its relative shape fixed and only the overall power amplitude scaled. The vertical axis is the largest stage-power ratio $\max_{i}P_{i}^{\mathrm{req}}/P_{\max,i}$, and the horizontal axis is the deadline tightness $\tau_{\min}^{P}/\tau_{\max}$, where $P_{\max,i}=V_{i}^{2}/\left(4r\right)$. The curve $I_{0}=\max_{i}I_{i}^{-}$ separates the demand-limited MSCD region from the single-current region. Above the curve, at least one stage has $I_{i}^{-}>I_{0}$, and, therefore, remains at its lower bound, while the other stages share $I_{0}$. Below the curve, $I_{0}>\max_{i}I_{i}^{-}$, so all stages share one current. The green demand-limited region is, therefore, the part of the parameter space where MSCD reduces heat relative to the peak-current benchmark.

Together, the two panels show the same KKT separation applied to two types of current bounds: the efficiency constraint sets upper bounds, while the load demands set lower bounds. In the following section, we focus on the load-constrained problem with prescribed stage powers $P_{i}^{\mathrm{req}}$ and use Equation (26) to construct the *Q*–τ Pareto front.

### 3.4. Internal Resistance and the Three-Dimensional Surface

Sweeping $\tau_{\max}$ over the feasible part of [10, 40]min in Equation (27) and substituting the result into Equation (28) traces the *Q*–τ Pareto front: the set of schedules for which heat cannot be reduced without increasing the discharge time [68]. Shorter deadlines force a larger $I_{0}$ and produce more heat; longer deadlines reduce $I_{0}$, so the schedule approaches the stage-wise lower bounds $I_{i}^{-}$ and the dissipation decreases. A knee point, therefore, provides a convenient representative compromise between time and heat. Normalizing discharging time and dissipation, we define

(36) $$x\left(\tau \right)=\frac{\tau -\tau_{\mathrm{scan}}^{\min}}{\tau_{\mathrm{scan}}^{\max}-\tau_{\mathrm{scan}}^{\min}},\;y\left(\tau \right)=\frac{Q\left(\tau \right)-Q_{\min}}{Q_{\max}-Q_{\min}},$$

where $\tau_{\mathrm{scan}}^{\min}$ and $\tau_{\mathrm{scan}}^{\max}$ are the lower and upper endpoints of the deadline scan, and $Q_{\min}$ and $Q_{\max}$ are the corresponding endpoint values of *Q*. The knee point is taken as the point of maximum perpendicular distance from the secant x+y=1 joining the two endpoints [69],

(37) $$\tau_{c}=\underset{\tau }{\arg\;\max}\frac{\left|x(\tau )+y(\tau )-1\right|}{\sqrt{2}}.$$

We now return to the five-stage example introduced in Section 3.2 and use it to quantify the knee point of the *Q*–τ Pareto front. Sweeping $\tau_{\max}$ over the feasible part of [10, 40]min in Equation (27) and substituting the resulting currents into Equation (28) gives the front. Applying the knee criterion gives $\tau_{c}≃21.94\;\min$, $Q_{c}≃10.75\;\mathrm{kJ}$, $\eta_{c}≃77.45\%$, and $I_{0}≃9.37\;A$. Other points on the Pareto front remain feasible and may be chosen when an application deliberately prioritizes speed or efficiency.

**Figure 6:**
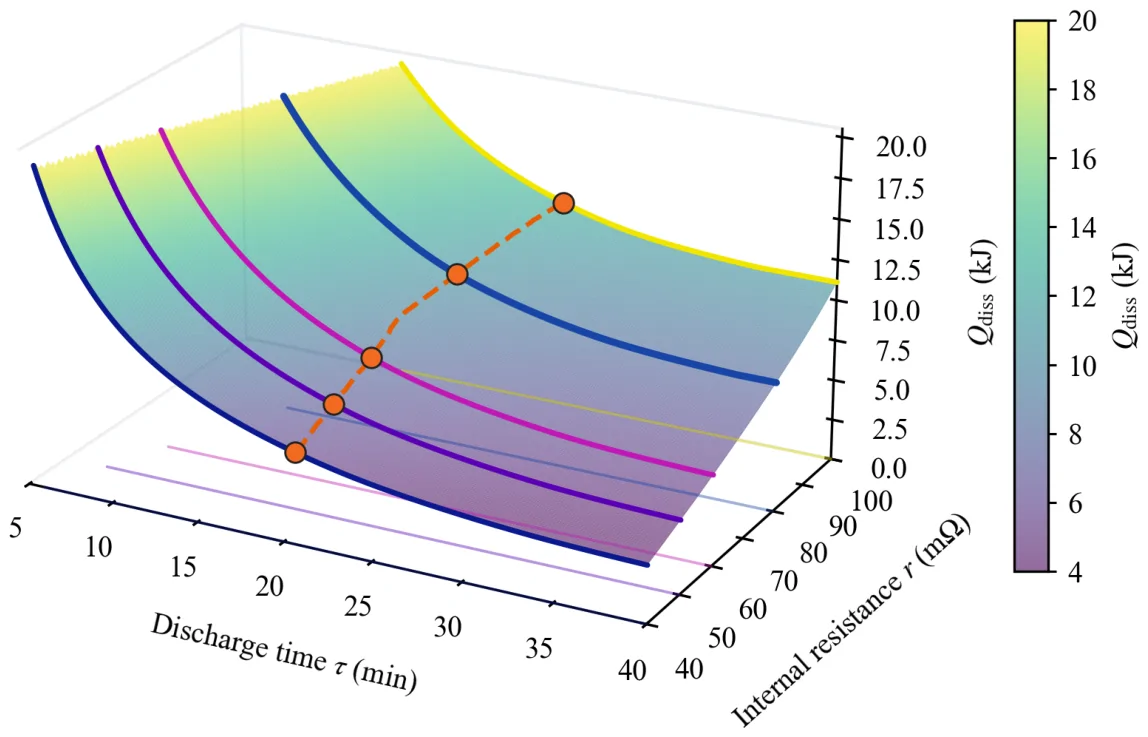
Three-dimensional Pareto surface $Q_{\mathrm{MSCD}}\left(\tau ,r\right)$ obtained by sweeping the deadline for r∈[40, 100] mΩ, using $P_{i}^{\mathrm{req}}=\left\{35.71,\;32.14,\;20.09,\;16.07,\;8.04\right\}\;W$. Color encodes $Q_{\mathrm{diss}}$. The dark-blue, purple, magenta, blue, and yellow solid curves show the constant-resistance Pareto fronts at r=40, 50, 60, 80, and 100mΩ, respectively. The orange dashed line and markers indicate the corner points of these Pareto fronts.

The preceding *Q*–τ Pareto front was obtained at a fixed internal resistance. Since the internal resistance varies among cells and can increase during ageing [52,70], we finally examine how internal resistance *r* reshapes the trade-off. Varying *r* over [0.04, 0.10]Ω and solving Equations (27) and (28) at each value, the surface $Q_{\mathrm{MSCD}}\left(\tau ,r\right)$ is obtained, as shown in Figure 6. The surface shows two trends. At fixed discharge time τ, a larger *r* produces greater Joule heat. Each two-dimensional slice has its own knee point on the *Q*–τ front for a fixed *r*. As *r* increases, this knee-point locus moves toward larger *Q* and larger τ, showing that a higher-resistance cell dissipates more heat when tight deadlines must be met. Reducing *r* lowers the whole surface and allows shorter deadlines to be reached with less dissipation.

## 4. Effect of Series Inductance

**Figure 7:**
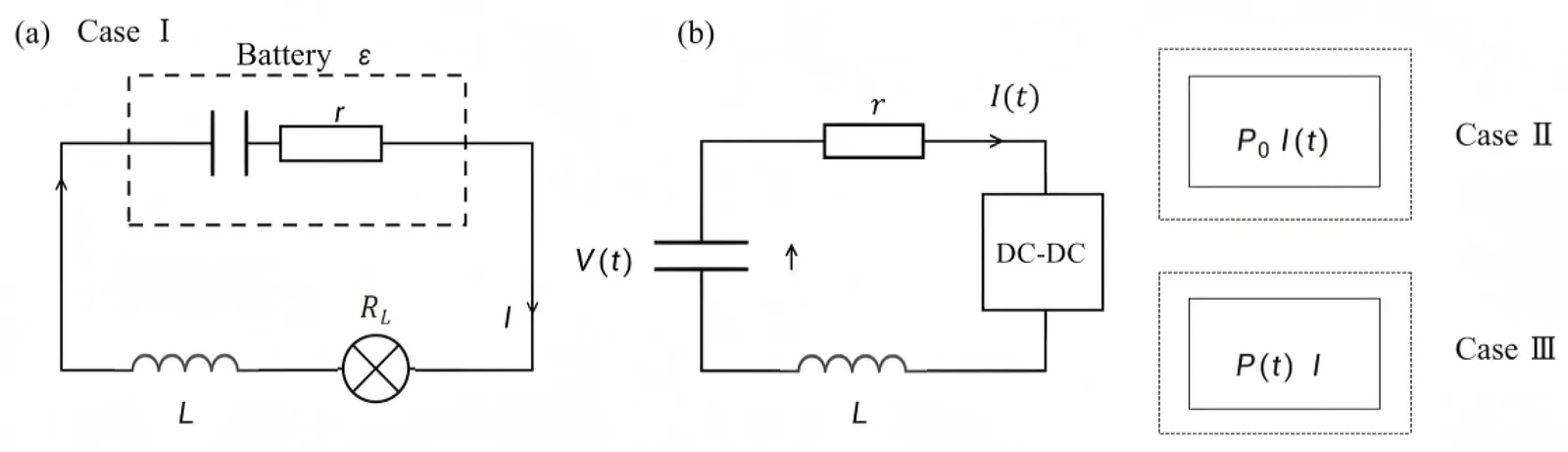
Equivalent circuits including an effective series inductance *L*. (**a**) Case I: a constant-EMF battery with internal resistance *r* and resistive load $R_{L}$ where $\varepsilon \equiv V_{0}$. (**b**) Cases II and III: a finite-capacitance battery connected to an ideal DC–DC converter. The converter maintains constant load power $P_{0}$ in Case II and constant current *I* in Case III.

The preceding analysis relied on resistance-dominated equivalent circuits to isolate the fundamental Ohmic-loss mechanism. To verify the generality of the resulting power–efficiency relations and the MSCD optimization, we introduce an effective series inductance *L* into the circuit (Figure 7). This inductance physically represents the high-frequency parasitic behaviors of cell tabs, cable connections, and test leads [71]. Because an ideal inductor exclusively stores and releases magnetic energy, it modulates the transient current dynamics and converter-voltage requirements without producing additional irreversible dissipation [58].

### 4.1. General Dynamic Power Balance

Across all three configurations in Figure 7, the instantaneous power balance incorporating the useful load power P(t) is modified to:

(38) $$V\left(t\right)I\left(t\right)=P\left(t\right)+rI^{2}\left(t\right)+LI\left(t\right)\dot{I}\left(t\right).$$

Here, the inductive term $LI\dot{I}=d\left(LI^{2}/2\right)/dt$ represents the conservative flux of magnetic energy rather than an additional irreversible loss. Consequently, the instantaneous efficiency η(t) and a dimensionless transient modulation factor α(t) are defined as:

(39) $$\eta \left(t\right)=\frac{P(t)}{V(t)I(t)}=1-\frac{rI(t)}{V(t)}-\frac{L\dot{I}\left(t\right)}{V(t)},\;\alpha \left(t\right)=1-\frac{L\dot{I}\left(t\right)}{V(t)}.$$

Eliminating the current I(t) yields the generalized dynamic envelope:

(40) $$P\left(t\right)=\frac{V^{2}\left(t\right)}{r}\eta \left(t\right)\left[\alpha (t)-\eta (t)\right].$$

Crucially, because α(t) depends dynamically on the current trajectory via $\dot{I}\left(t\right)$, Equation (40) is no longer a closed algebraic relation. Specifying the transient power–efficiency trade-off and its maximum-power condition now strictly requires a predefined load model or converter-control law.

### 4.2. Implications for the Three Cases

For Case I, the battery drives a fixed resistive load $R_{L}$; thus, the current relaxes exponentially with the characteristic time $\tau_{L}=L/\left(r+R_{L}\right)$. During the transient phase, the output power follows $P\left(t\right)=V_{0}^{2}\eta^{2}\left(t\right)/R_{L}$, which is fixed by the load resistance. Therefore, the inductance only determines the evolution rate of η(t) without influencing the instantaneous algebraic relation. After the transient decays and the inductive voltage vanishes, the steady-state parabola $P_{\infty }=V_{0}^{2}\eta_{\infty }\left(1-\eta_{\infty }\right)/r$ is recovered, leaving the maximum-power efficiency $\eta_{\mathrm{EMP}}=1/2$ unchanged. For Case II, the battery supplies a constant load power $P\left(t\right)=P_{0}$. The finite capacitance imposes the kinematic constraint $C\dot{V}=-I$, while the inclusion of *L* elevates the current to an independent dynamic state variable. Consequently, the generalized envelope expands into an exact differential constraint:

(41) $$\frac{L}{CV^{2}}P_{0}^{2}+\left(r\eta -L\dot{\eta }\right)P_{0}-\eta^{2}V^{2}\left(1-\eta \right)=0.$$

When the inductive transients decay (L→0 or $\dot{\eta }\to 0$), this seamlessly reduces to the purely algebraic baseline relation:

(42) $$P_{0}=\frac{V^{2}}{r}\eta \left(1-\eta \right).$$

Thus, inductance replaces the algebraic trade-off with a differential one during transients. Furthermore, a local stability analysis reveals that under an ideal constant-power demand, the high-efficiency (low-current) operating branch is dynamically unstable and must be actively stabilized by the converter. For Case III, the battery operates under the multistage constant-current protocol, meaning $\dot{I}=0$ during each bulk discharging stage. Therefore, the internal stage-wise power–efficiency relations remain entirely unaffected. Inductive effects are strictly confined to the finite switching intervals between stages. For a transition *i* of duration $\delta_{i}$ with a current step $\Delta I_{i}=I_{i+1}-I_{i}$, the transient penalties scale as:

(43) $$q_{i}^{\mathrm{sw}}=O\left(\delta_{i}\right),\;Q_{i}^{\mathrm{sw}}=O\left(\delta_{i}\right),\;L\dot{I}=O\left(\frac{L|\Delta I_{i}|}{\delta_{i}}\right),$$

where $q_{i}^{\mathrm{sw}}$ and $Q_{i}^{\mathrm{sw}}$ are the transition charge and Joule heat. Because a rapid current transition induces a large voltage spike $L\dot{I}$, the hardware’s converter-voltage limit establishes a strict lower bound on $\delta_{i}$. Provided $\delta_{i}$ remains much shorter than the bulk stage duration $T_{i}$, these switching penalties are negligible, robustly preserving the resistance-only MSCD constraints, dissipation functional, and KKT solution. These results demonstrate that series inductance governs switching transients, converter-voltage limits, and local stability, but leaves the fundamental steady-state and constant-current thermodynamic bounds mathematically intact. Rigorous derivations, stability proofs, and quantitative switching corrections are detailed in Appendix A.

## 5. Summary and Outlook

In this paper, we investigated battery discharging from a finite-time thermodynamic perspective. By evaluating three representative models—constant electromotive force, finite-capacitance RC dynamics, and active constant-current control—we identified a universal parabolic power–efficiency envelope, P∝η(1−η). Across all models, the efficiency at maximum power strictly bounds to one half, serving as the exact electrochemical counterpart to the half-Carnot limit [26]. We further demonstrated that introducing a series inductance only modifies the transient power–efficiency dynamics and converter-voltage requirements, leaving the steady-state parabola and constant-current stage relations strictly intact. Consequently, the resistance-only MSCD formulation is seamlessly recovered provided that the switching intervals are short and remain feasible under converter-voltage limits. Building upon these robust bounds, we formulated the multistage constant-discharging (MSCD) optimization under stage-wise load demands and a global deadline. The Karush–Kuhn–Tucker (KKT) conditions yielded a remarkably compact optimal policy: $I_{i}^{★}=\max\left(I_{i}^{\mathrm{req}},\;I_{0}\right)$, where the uniform baseline $I_{0}$ is fixed by the deadline. Conversely, a minimum instantaneous-efficiency constraint yielded the dual upper-bound structure $I_{i}^{★}=\min\left(I_{c},\;I_{i}^{\eta }\right)$. These complementary solutions successfully extend the multistage charging framework [42,43,44,45] into the load-driven discharging regime.

For the load-constrained MSCD problem, sweeping the global deadline traces a robust dissipation–time (*Q*–τ) Pareto front [68,69]. As demonstrated in our PHEV analysis, the MSCD strategy significantly mitigated Joule heating compared to peak-current benchmarks. We also quantified how increasing the internal resistance globally shifts this operational boundary, elevating the dissipated heat and pushing the Pareto knee point toward longer discharging times. Crucially, the current study, together with our previous work on optimal charging [42], establishes a complete and unified finite-time thermodynamic framework for the entire battery charge–discharge cycle. This comprehensive paradigm paves the way for analyzing multidimensional performance trade-offs in practical energy storage.

Future work will bridge the gap between these idealized thermodynamic bounds and practical applications through several realistic extensions. First, incorporating more detailed circuit architectures and heat-generation mechanisms—such as higher-order RC/Thevenin/Randles elements and entropy-change heat from electrode reactions [63]—will rigorously test the robustness of the proposed power–efficiency bound beyond the minimal equivalent circuit. Second, relaxing the assumption of a constant internal resistance by formulating it dynamically as a function of temperature and state of charge (SOC) will naturally couple the discharging dynamics to a rigorous thermal balance framework [72,73]. Furthermore, the analysis can be enriched by integrating advanced electrochemical models to capture internal concentration gradients [60,61,62], alongside long-term degradation costs as competing optimization objectives [52,70]. These extensions will directly empower the scheduling layer of modern Battery Management Systems while preserving the elegantly compact KKT current-bound structure established here.

## Figures and Tables

**Figure A1:**
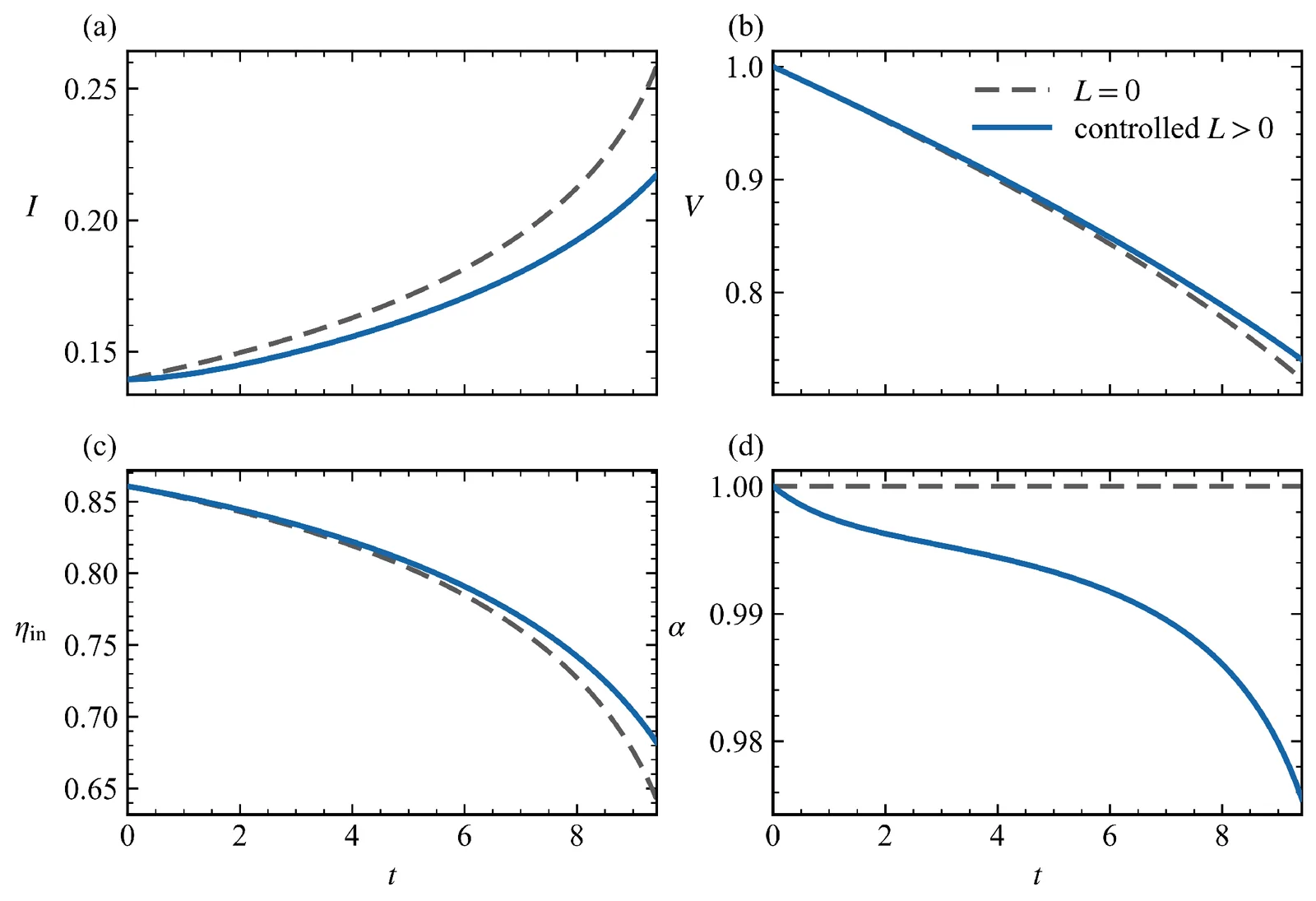
Controlled tracking of the low-current branch $I_{-}\left(V\right)$: (**a**) current, (**b**) source voltage, (**c**) cell-to-converter input power ratio $\eta_{\mathrm{in}}$, and (**d**) transient factor $\alpha =1-L\dot{I}/V$. Dashed and solid curves denote the resistance-only limit L=0 and the controlled inductive trajectory L>0, respectively.

## Data Availability

The original contributions presented in this study are included in the article. Further inquiries can be directed to the corresponding author.

## Appendix A. Detailed Analysis of Series Inductance

This appendix details the derivations, stability proofs, numerical validations, and MSCD switching corrections supporting Section 4. The overarching dynamic power balance is given by Equation (38).

### Appendix A.1. Case I: Transient and Steady-State Relations

For a constant-EMF source $V_{0}$ driving a resistive load $R_{L}$, the circuit equation $L\dot{I}+\left(r+R_{L}\right)I=V_{0}$ yields the exponential relaxation

(A1) $$I\left(t\right)=I_{\infty }+\left[I\left(0\right)-I_{\infty }\right]e^{-t/\tau_{L}},$$

where I(0) is the initial current, $I_{\infty }=V_{0}/\left(r+R_{L}\right)$ is the steady-state current, and $\tau_{L}=L/\left(r+R_{L}\right)$ is the inductive relaxation time. For a fixed $R_{L}$, the instantaneous power and efficiency are $P\left(t\right)=R_{L}I^{2}\left(t\right)$ and $\eta \left(t\right)=R_{L}I\left(t\right)/V_{0}$. Eliminating I(t) directly reveals

(A2) $$P\left(t\right)=\frac{V_{0}^{2}}{R_{L}}\eta^{2}\left(t\right).$$

Thus, *L* merely dictates the temporal relaxation rate $\tau_{L}$, leaving the geometric trajectory of the transient power–efficiency relation completely unaltered. As t→∞, the inductive voltage $L\dot{I}\to 0$. Eliminating $R_{L}$ from the asymptotic states seamlessly recovers the steady-state parabola:

(A3) $$P_{\infty }=\frac{V_{0}^{2}}{r}\eta_{\infty }\left(1-\eta_{\infty }\right).$$

### Appendix A.2. Case II: Differential Constraint and Local Stability

Under the finite-capacitance kinematic constraint $C\dot{V}=-I$, substituting I=P/(ηV) into Equation (38) elevates I(t) to an independent dynamic state:

(A4) $$\dot{\eta }=\eta \frac{\dot{P}}{P}+\frac{P}{CV^{2}}+\frac{r\eta }{L}-\frac{\eta^{2}V^{2}\left(1-\eta \right)}{LP},\;\dot{V}=-\frac{P}{C\eta V}.$$

For a strictly constant load power $P\left(t\right)=P_{0}$, the system simplifies to the exact differential constraint

(A5) $$\frac{L}{CV^{2}}P_{0}^{2}+\left(r\eta -L\dot{\eta }\right)P_{0}-\eta^{2}V^{2}\left(1-\eta \right)=0,$$

which smoothly degenerates to the algebraic baseline $P_{0}=\frac{V^{2}}{r}\eta \left(1-\eta \right)$ as L→0. Crucially, the total energy balance satisfies

(A6) $$\frac{d}{dt}\left(\frac{1}{2}CV^{2}+\frac{1}{2}LI^{2}\right)=-P\left(t\right)-rI^{2},$$

confirming that inductance exclusively governs reversible magnetic-energy storage without injecting additional irreversible dissipation.

To evaluate local stability under $P\left(t\right)=P_{0}$, we fix *V* at its instantaneous value. The current subsystem admits two stationary roots:

(A7) $$I_{\pm }\left(V\right)=\frac{V\pm \sqrt{V^{2}-4rP_{0}}}{2r},\;0<P_{0}<\frac{V^{2}}{4r}.$$

Writing the current dynamics as $\dot{I}=f\left(I;V\right)=-\frac{r}{LI}\left(I-I_{-}\right)\left(I-I_{+}\right)$ yields the Jacobians

(A8) $$f^{\prime }\left(I_{-}\right)=\frac{\sqrt{V^{2}-4rP_{0}}}{LI_{-}}>0,\;f^{\prime }\left(I_{+}\right)=-\frac{\sqrt{V^{2}-4rP_{0}}}{LI_{+}}<0.$$

Since $f^{\prime }\left(I_{-}\right)>0$, the high-efficiency (low-current) branch $I_{-}$ is locally dynamically unstable under an ideal constant-power demand. Consequently, practical operation along this efficient branch must be actively stabilized by the converter.

### Appendix A.3. Numerical Illustration of Controlled Active Tracking

To demonstrate this active stabilization, we introduce a proportional controller tracking the optimal reference $I_{\mathrm{ref}}\left(V\right)=I_{-}\left(V\right)$:

(A9) $$L\dot{I}=-\kappa \left[I-I_{\mathrm{ref}}\left(V\right)\right],\;C\dot{V}=-I,\;\kappa >0.$$

The converter-input voltage, power, and efficiency ratio become

(A10) $$U_{\mathrm{in}}=V-rI-L\dot{I},\;P_{\mathrm{in}}=U_{\mathrm{in}}I,\;\eta_{\mathrm{in}}=\frac{P_{\mathrm{in}}}{VI}.$$

Assuming an ideal lossless converter equipped with an energy buffer $E_{\mathrm{dc}}$, the external load can rigidly maintain $P_{0}$ by absorbing the transient tracking mismatch ${\dot{E}}_{\mathrm{dc}}=P_{\mathrm{in}}-P_{0}$, yielding the global balance $\frac{d}{dt}\left(\frac{1}{2}CV^{2}+\frac{1}{2}LI^{2}+E_{\mathrm{dc}}\right)=-P_{0}-rI^{2}$.

Figure A1 compares the resistance-only trajectory (L=0) against the controlled inductive trajectory (L>0) using the dimensionless parameters V(0)=1, r=1, C=6, and $P_{0}=0.12$ and deliberately large inductive/gain terms L=0.8,κ=0.8 to amplify visibility. As *V* decays, the inductive lag confines the controlled current strictly below the L=0 limit, decelerating the source voltage drop. Because $\dot{I}>0$, magnetic energy accumulates, locking the transient factor $\alpha =1-L\dot{I}/V$ firmly below unity. (For this specific illustrative configuration, the maximum tracking error is $\max_{t}\left|I-I_{\mathrm{ref}}\right|=2.27\times 10^{-2}$, with a peak input-power mismatch of 8.64%.)

### Appendix A.4. Case III: MSCD Switching Corrections and KKT Robustness

During the internal constant-current stages ($\dot{I}=0$), the resistance-only relations perfectly govern the MSCD protocol:

(A11) $$P_{i}\left(t\right)=V\left(t\right)I_{i}-rI_{i}^{2},\;\eta_{i}\left(t\right)=1-\frac{rI_{i}}{V(t)}.$$

Inductive penalties exclusively arise during the finite switching intervals between stages. To analytically quantify this, consider a smooth cubic transition of duration $\delta_{i}$ linking $I_{i}$ and $I_{i+1}$:

(A12) $$I\left(t\right)=I_{i}+\Delta I_{i}\left(3x^{2}-2x^{3}\right),\;x=\frac{t-t_{i}}{\delta_{i}},$$

which enforces $\dot{I}\left(t\right)=\frac{6\Delta I_{i}}{\delta_{i}}x\left(1-x\right)$ and smoothly matching boundary conditions. Integrating Equation (A12) over time yields the battery voltage V(t), and Kirchhoff’s voltage law then determines the converter-input voltage $U_{\mathrm{in}}\left(t\right)$. The switching power $P_{\mathrm{sw}}\left(t\right)$ and instantaneous efficiency $\eta_{\mathrm{sw}}\left(t\right)$ are subsequently evaluated from the same definitions used above, with the efficiency including both ohmic and inductive voltage drops.

Assuming ideal lossless conversion, transition *i* remains physically feasible strictly if $U_{\min}\leq U_{\mathrm{in}}\left(t\right)\leq U_{\max}$ and $P_{\mathrm{sw}}\left(t\right)\geq P_{\mathrm{req}}\left(t\right)$. Since the peak inductive spike scales as $\max_{t}|\dot{I}\left(t\right)|=3|\Delta I_{i}|/\left(2\delta_{i}\right)$, the absolute hardware converter-voltage limits dictate the minimum allowable switching time $\delta_{i}$.

Integrating the cubic profile extracts the transferred charge $q_{i}^{\mathrm{sw}}$ and root-mean-square switching current $I_{i,\mathrm{rms}}^{\mathrm{sw}}$:

(A13) $$q_{i}^{\mathrm{sw}}=\frac{\delta_{i}}{2}\left(I_{i}+I_{i+1}\right),\;{\left(I_{i,\mathrm{rms}}^{\mathrm{sw}}\right)}^{2}=\frac{13I_{i}^{2}+9I_{i}I_{i+1}+13I_{i+1}^{2}}{35}.$$

The switching Joule heat and magnetic-energy shift directly follow as $Q_{i}^{\mathrm{sw}}=r{\left(I_{i,\mathrm{rms}}^{\mathrm{sw}}\right)}^{2}\delta_{i}$ and $\Delta E_{L,i}=\frac{L}{2}\left(I_{i+1}^{2}-I_{i}^{2}\right)$. Regardless of the precise profile shape, any smooth transition yields the rigorous scaling:

(A14) $$q_{i}^{\mathrm{sw}}=O\left(\delta_{i}\right),\;Q_{i}^{\mathrm{sw}}=O\left(\delta_{i}\right),\;L\dot{I}=O\left(\frac{L|\Delta I_{i}|}{\delta_{i}}\right).$$

Accounting for these N−1 finite transitions explicitly modifies the global MSCD Joule heat objective, deadline, and charge constraints:

(A15) $$\begin{array}{cc} Q_{\mathrm{tot}} & =r\sum_{i=1}^{N}I_{i}^{2}T_{i}+\sum_{i=1}^{N-1}Q_{i}^{\mathrm{sw}}, \end{array}$$

(A16) $$\begin{array}{cc} \tau_{\max} & \geq \sum_{i=1}^{N}T_{i}+\sum_{i=1}^{N-1}\delta_{i}, \end{array}$$

(A17) $$\begin{array}{cc} C(V_{0}-V_{f}) & =\sum_{i=1}^{N}I_{i}T_{i}+\sum_{i=1}^{N-1}q_{i}^{\mathrm{sw}}. \end{array}$$

Because $q_{i}^{\mathrm{sw}}$ and $Q_{i}^{\mathrm{sw}}$ dynamically couple adjacent stages $I_{i}$ and $I_{i+1}$, the idealized stage-wise KKT decoupling $I_{i}^{★}=\max\left(I_{i}^{\mathrm{req}},I_{0}\right)$ is no longer strictly exact. However, provided the switching intervals remain brief relative to the bulk stages ($\delta_{i}\ll T_{i}$) and feasible under converter limits, the switching penalties are completely overshadowed: $\sum q_{i}^{\mathrm{sw}}\ll \sum I_{i}T_{i}$ and $\sum Q_{i}^{\mathrm{sw}}\ll r\sum I_{i}^{2}T_{i}$. Under this practical operational limit, the finite-switching corrections vanish, robustly restoring the exact resistance-only MSCD optimization structure.
